# Lymphoid Interstitial Pneumonia in Common Variable Immune Deficiency – Case Report With Disease Monitoring in Various Therapeutic Options: Pleiotropic Effects of Rituximab Regimens

**DOI:** 10.3389/fphar.2018.01559

**Published:** 2019-01-18

**Authors:** Przemyslaw Zdziarski, Andrzej Gamian

**Affiliations:** ^1^Department of Immunology of Infectious Diseases, Ludwik Hirszfeld Institute of Immunology and Experimental Therapy, Polish Academy of Sciences, Wrocław, Poland; ^2^Department of Clinical Immunology, Lower Silesian Center, Wrocław, Poland; ^3^Military Institute WITI Wrocław, Wrocław, Poland

**Keywords:** lymphoid interstitial pneumonia (LIP), lymphoproliferative disease (LPD), rituximab, innate immune response, natural killer (NK) natural killer T-cells (NKT), FoxP3+ regulatory T cells (Treg), immune dysregulation, common variable immune deficiency (CVID)

## Abstract

Lymphoid interstitial pneumonia (LIP) is a rare lymphoproliferative disease. LIP in common variable immunodeficiency (CVID) was observed in a patient during immunomodulatory therapy after progression of the disease (i.e., glucocorticoids, immunoglobulin dose escalation, and finally rituximab). Due to humoral immunodeficiency and serious serum sickness rituximab was used initially at a low dose (150 mg/m^2^ weekly). It resulted in temporary remission with the decrease of serum paraproteinemia, β_2_-microglobulin (β2M) and SUV decrease as well as increase of FVC. Owing to the relapse after 6-month remission in the second cycle a standard dose of rituximab was used (375 mg/m^2^). Therapeutic regimen with 375 mg/m^2^ of Rtx in optimal schedule (i.e., every 3 weeks) resulted in no longer remission but higher incidence of opportunistic infections. Finally, after another cycle of immunotherapy FVC, paraproteinemia and β2M level normalization were observed as well as the decrease of severe splenomegaly. In laboratory and immunological progress the increase of NK and NKT cells was observed after the initial dose but the standard one caused NK cell increase only. Unfortunately, the decrease of CD19+Bcells was comparable between both doses, as was the decline of FoxP3+ regulatory T cell. On the contrary, after the low dose absolute T cell (both CD4 and CD8) number decreased but after the standard one – it normalized. Rtx (especially in low dose) brought further increase of persistent T cell activation (CD38+ T cells made up 79%). Innate immune response and the decrease of Treg are a compensatory pathways for the decrease of B and T cells. Immunodeficiency requires a different investigative approach to a immunotherapy.

**Clinical Trial Registration:** ClinicalTrials.gov, NCT02789397.

## Introduction

Lymphoid interstitial pneumonia (LIP) is a rare restrictive disease that usually coexists with immunodeficiency (Hurst et al., 2017; Zdziarski et al., 2017), classified as LPD. The natural history and prognosis of LIP are poorly understood. Contrary to low incidence in general population LIP is the most common cause of pulmonary disease after *Pneumocystis jirovecii* pneumonia (PCP) in HIV-positive children. Serum protein electrophoresis in laboratory testing are done because about 80% of patients have a serum protein abnormality, most commonly a polyclonal gammopathy and hypogammaglobulinemia. Noteworthy, polyclonal IgM-paraproteinemia, massive splenomegaly, lymphadenopathy, pulmonary infiltration coincides with non-random Ig gene rearrangement (narrow B cell receptor repertoire) and regulatory T cells decrease as leading parameter of LIP (i.e., corresponding with Ki-67 and histological findings) (Zdziarski et al., 2017). Although LIP diagnosis and criteria are well described, the narrow therapeutic regimen is still an important problem in clinical practice, this is because LIP incidence is low, patients are not a homogenous group and a prospective cohort study is very difficult (LIP affects <1% of adults with immunodeficiency or with HIV infection, clinical trial NCT02789397 is still open (first posted : June 3, 2016 Last update posted : March 21, 2018). It is therefore not surprising that in recent years there has not been any increase in the number of investigations of a new therapeutic regimen.

As regards first-line treatment with corticosteroids alone and immunoglobulin therapy modification there was no consensus (Hurst et al., 2017). The latest therapeutic regimen consists of rituximab and cytotoxic drugs, mycophenolate (Chase et al., 2013; Jolles et al., 2017), azathioprine (Vitale et al., 2015) or 6-mercaptopurine (Chase et al., 2013). It is controversial in severe immunodeficiency due to the risk of opportunistic infections (e.g., fungal) during prolonged use. Lack of EBV-specific CD8 T cells and CMV-induced lymphoproliferative process exacerbation described previously also spotlights infectious issues (Zdziarski et al., 2017). Furthermore, during and following treatment with standard (375 mg/m^2^) dose of rituximab PCP prophylaxis is recommended for patients with other types of granulomatous interstitial lung disease, i.e., granulomatosis with polyangiitis or microscopic polyangiitis (product characteristics of MabThera^®^^1^). Quite a few therapeutic interventions have been tried in small populations of patients with variable effect and variable tolerance of immunosuppressive therapy (Chase et al., 2013). Until now, rituximab monotherapy has not been thoroughly described: only in a personal communication (Chase et al., 2013) or in a case report in Sjogren’s syndrome (Swartz and Vivino, 2011; Tansy and McLean-Tooke, 2013). In the therapy of CVID-induced LIP only one observation was published, but unfortunately with a standard dose and in combination with azathioprine (Vitale et al., 2015). The same model is used in the current clinical trial (NCT02789397) in 18-month-long period. Although no dose reductions of rituximab are recommended in therapeutic regimen of other LPDs, i.e., chronic lymphocytic leukemia (CLL) or NHL, combined immunodeficiency such as common variable immunodeficiency (CVID) suggests caution. Furthermore, there are numerous reports stating that rituximab may induce serious pneumonitis (Lioté et al., 2010).

Herein, we report a case of progressive, refractory LIP which was successfully put into sustained complete remission with a low dose rituximab (150 mg/m^2^), monitored with dysproteinemia, FoxP3+Treg, β_2_-microglobulin (β2M) level, spleen size, and SUV. For comparison the leading parameters are used in therapeutic drug monitoring during therapy with standard (375 mg/m^2^) dose of rituximab, usually used in NHL.

## Case Report

**FIGURE 1:**
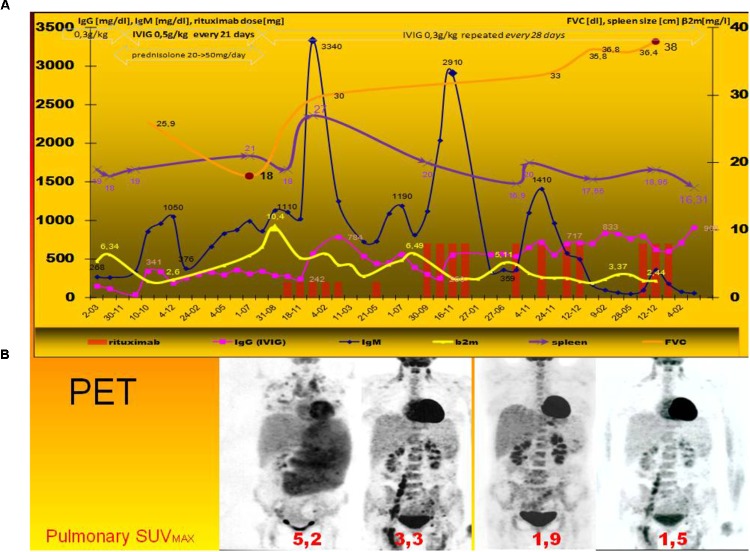
Clinical course of rituximab monotherapy in lymphoproliferative disease exacerbation. Timeline of treatment of LIP with low and standard dose of rituximab following the typical therapeutic regimen (i.e., escalating dose of prednisolone and intravenous immunoglobulin) (Hurst et al., 2017). Figure show 5-year-long period, rituximab monotherapy, and PET findings contrary to clinical trial NCT02789397^2^). **(A)** Quantitative and functional evaluation of leading parameters of lymphoid interstitial pneumonia (2): IgM level, β_2_-microglobulin (β2m), spleen size, and pulmonary restriction (FVC) were presented. All spirometric measurements were performed according to the recommendations of the European Respiratory Society. Prednisolone therapy and immunoglobulin dosage adjustment (to 0.5 g/kg repeated every 21 days) were ineffective. Progression of the disease corresponds with high β_2_-microglobulin and very high level of non-monoclonal IgM paraproteinemia, spleen size, but without typical signs of malignancy. After corticosteroids withdrawal and rituximab monotherapy fast and significant increase of forced vital capacity (FVC) was observed as well as decrease of splenomegaly. After rituximab therapy IgM – paraproteinemia, the sign of lymphoproliferative disease, pulmonary restrictive disease resolved. The clinical improvement corresponds with less IgG consumption/IVIG requirement. **(B)** PET imaging of therapeutic response in LIP (Jolles et al., 2017). Bottom panel shows corresponding PET-tomography before, after the first (150 mg/m^2^), second and third course (both-375 mg/m^2^) of rituximab monotherapy. The maximal standardized uptake value (SUV) in pulmonary granulomas are shown. Before therapy the highest uptake of (18)F-fluoro-deoxyglucose was seen in spleen and pulmonary granulomas with lymphoid tissue: SUV was shown. PET.PET findings are useful (Zdziarski et al., 2017) and more representative than high-resolution CT scans of the chest (data not shown).

A CVID-diagnosed, 25-year-old, non-smoker woman was admitted to our center with LIP progression: CVID diagnosis was consistent with ESID criteria. The restrictive, granulomatous lung disease developed: open lung biopsy and histological examination showed lymphocytic infiltration of interstitial tissue: LIP diagnosis was confirmed by the histologic examination as well as T and B cells repertoire analysis as described previously (Zdziarski et al., 2017). Before the therapy spleen extended to the iliac crest (27 cm, see Figure 1 bottom panel): subileus was observed due to the pressure on intestines.

### Laboratory Investigations

Progressive LPD was observed with hyperviscosity, paraproteinemia, high β2M and IgM level (Figure 1 upper panel). Respiratory functional study showed: reduced forced vital capacity (FVC-60% of the predicted volume, i.e., 23 dl) (Miller et al., 2005) and low diffusion capacity (for carbon monoxide DLCO-5,18 mmol/min/Kpa, i.e., 49%). BAL, blood, urine, bone marrow, sputum cultures, and MALDI analyses were all free of bacteria, mycobacteria, actinobacteria, and fungi. Analysis of fluid obtained by BAL showed an increase in the total cell count, predominantly in neutrophils and lymphocytes but without significant predominance of NKT cells as observed in hypersensitivity pneumonitis or pulmonary sarcoidosis (Katchar et al., 2005; Korosec et al., 2007) (data not shown). Initial immunoparameters and cytometric analysis are shown in the first column of Table 1.

**Table 1:** Immunoparameters and cytometric analysis of peripheral lymphocytes during rituximab monotherapy at low and standard dose.

(% Peripheral lymphocytes)	First exacerbation (before rituximab)	After 150 mg/m^2^	After 350 mg/m^2^
CD19	4,7	2,3	1
Anti-Kappa+CD19+	0,07	0,01	0,00
Anti-Lambda+CD19+	0,05	0,00	0,00
CD19+38+	2,2	0,7	0,1
CD19+138+	0,01	0,00	0,00
FoxP3+CD4+CD25high	54/100 μl	20/100 μl	6/100 μl
CD38+T cells	21,7% (196/μl)	79,8% (828/μl)	43,3% (319/μl)
NK/NKT ratio (initial-2,7)	0,37	1,38	0,26
EBV BMLF-1 (pentamer HLA-A^∗^0201/GLCTLVAML)	0,02	0,06	NT^1^
CMV pp65 (pentamer HLA-A^∗^0201/NLVPMVATV)	2,21	1,83	NT^1^
LMP (pentamer HLA-A^∗^0201/CLGGLLTMV)	0,05	0,17	NT^1^
CD8+EBV BMLF-1+	0,02	0,16	NT^1^
CD8+CD57+EBV BMLF-1+	0,01	0,04	NT^1^
CD8high+EBV BMLF-1+	0,00	0,02	NT^1^
CD8high+CD57+EBV BMLF-1+	0,00	0,02	NT^1^
CD8+LMP+	0,02	0,15	NT^1^
CD8+CD57+LMP+	0,02	0,05	NT^1^
CD8high+LMP-1+	0,06	0,03	NT^1^
CD8high+CD57+LMP+	0,01	0,03	NT^1^
CD8+CMV pp65+	2,18	1,65	NT^1^
CD8+CD57+CMV pp65+	2,03	1,45	NT^1^
CD8high+CMV pp65+	10,90	1,24	NT^1^
CD8high+CD57+CMV pp65+	1,81	1,64	NT^1^
Quantiferon [IU/ml]			
CMV-specific epitopic peptides	9,06	1,92	0,90
Control	0,66	0,08	0,90
PHA	4,00	4,71	2,48


**FIGURE 2:**
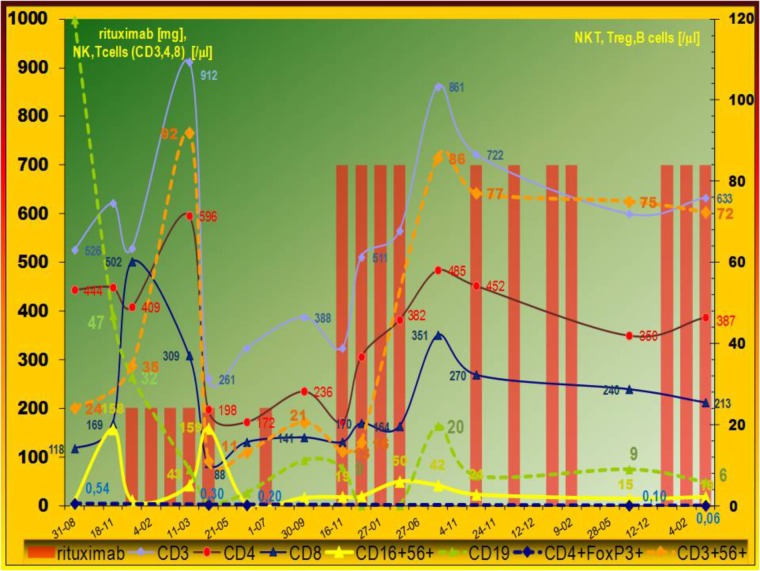
Evolution of peripheral lymphocytes populations. Immunomodulatory effect of rituximab on cellular compartment. Pleiotropic influence of low (150 mg/m^2^) rituximab dose. Data expressed as absolute numbers per μl. The cell counts were analyzed during LIP exacerbation – multiorgan lymphoproliferative disease development. Typical low level of invariant natural killer T (NKT), natural killer (NK), and regulatory T cells (Treg) was observed. After rituximab therapy abnormal innate immunity – absolute number of NK and NKT cells increased, but gradual decrease of FoxP3+ regulatory T cells was observed with increase activated CD38+T cells (not shown). Leukocyte counts analyses were done by the Sysmex Automated Hematology System. Flow cytometry was performed using a FACS Calibur flow cytometer (Becton Dickinson) and a count of lymphocyte subset was calculated by the frequency multiply the lymphocyte counts.

*Although B cell count was normal (72–120 as shown in Figure 2), most of them were surface light chain negative. CTL was tested for specificity using pentamer technique: vigorous CD8 response to CMV antigens was normalized after rituximab therapy. It corresponds with CMV-induced gamma interferon-releasing assay (Quantiferon). Physiologically absolute NK and CD3+56+ natural killer T cells (NKT) number and percentage are comparable but here patients during exacerbation show significant decrease of high Nk level. Gradual decrease of FoxP3+ regulatory T cells was observed. ^*1*^Significant decrease below detection limit (0.01%) was observed. BMLF1, BamHI-M leftward reading frame 1 (immediate-early nuclear EBV antigen); LMP1, EBV latent membrane protein 1; Quantiferon, gamma interferon-releasing assay.*

### Relevant Therapeutic Interventions and Their Outcomes

Contrary to previous data (Arish et al., 2007), after intravenous immunoglobulin (IVIG) dosage adjustment (from 0.3 to 0.5 g/kg every 21 days, accordingly to the decrease of IgG before replacement) was ineffective as well as glucocorticoids (topical, then systemic with high-dose methylprednisolone up to 50 mg/daily) (Figure 1).

Due to serum sickness with high IgM-paraproteinemia and high risk opportunistic infections, especially EBV reactivation (see pentamer analysis in Table 1) lower rituximab dose (150 mg/m^2^ every week) was used. The patient did not receive other concomitant medication. The sixth dose (Figures 1, 2) was delayed due to spontaneously resolved temporary neurological sign.

After temporary (6-month) remission and relapse the standard dose (375 mg/m^2^) infusion every 21 days) was used with the same remission interval (about 6 months). Such optimal schedule for RTx dosage every 3 weeks was described elsewhere (Golay et al., 2013). In the third course patients could not receive regular regimen because of the increased incidence of infectious processes (especially pneumococcal, herpes zoster reactivation).

Relevant data from episode of care is shown as a timeline (Figure 1) and in Table 1.

Unexpectedly, after rituximab therapy the LPD was stopped (Figure 1B, as shown in PET). Restrictive pulmonary disease and FVC systematically improved and finally normalized (Figure 1A). The decrease of spleen size and leading biochemical and serological parameters were observed (i.e., β2M and IgM level, see Figures 1, 2) in line with LIP regression and FVC increase. Fortunately, rituximab therapy was a glaring alternative for IVIG escalation: It also reduced IgG requirements by up to 40% of adjustment dose (Figure 1A). The serological and clinical effectiveness (IgM, FVC and PET finding) of both schedules were comparable, but more significant reduction of β2M was observed after 150 mg/m^2^ of rituximab (Figure 1A).

Parallel cytometric lymphocyte analysis is organized as a timeline (Figure 2). A low dose does not affect the incidence of infections, but surprisingly it shows more pleiotropic immunomodulatory effects. There was observed high increase in low absolute number of CD16+56+ NK cells. Interestingly, T cell (CD4 and CD8) level also decreased, but it increased after the standard dose without the considerable increase of very low level of regulatory FoxP3 positive T lymphocytes (Treg) (Table 1). Absolute number of natural killer T cells (NKT, CD3+56+) increased temporarily after low and standard dose (Figure 2). Therefore, initial NK/NKT ratio (2.7) decreased during LPD progression, but it increased after the initial low dose of rituximab (Table 1). Rituximab therapy shows a significant inhibitory effect on CMV-specific CD8+ cytotoxic T lymphocyte (CTL) levels and interferon gamma release, but RTx-induced CD38 expression on T cells was higher after the low dose (Table 1). Interestingly, a low dose causes increase of post-mitogenic (PHA) interferon release.

## Discussion

Lymphoid interstitial pneumonia is lymphocytic infiltration of the alveolar interstitium and air spaces, i.e., an extreme manifestation of lymphoid tissue reaction in the lung.

Evidence of an immunodysregulation and autoimmune etiology includes its frequent association with Sjögren syndrome (25% of cases of LIP) and other disorders (e.g., SLE, RA, Hashimoto thyroiditis-14% of cases). Evidence of an indirect viral etiology includes frequent association with immunodeficient states, especially CMV (Zdziarski et al., 2017). CVID is the most common symptomatic primary immunodeficiency characterized by defective B cell function, reduced levels of all major immunoglobulin classes and recurrent infections (Chapel et al., 2008; Hurst et al., 2017). Reduced B cell number is sometimes observed. Our observation corresponds with the latest findings that show substantial perturbations in innate (NKT) and adoptive (CD8, Treg cells) cellular immune response in CVID (Paquin-Proulx et al., 2013). Such perturbations have not been tested nor described in LIP, contrary to significant predominance of NKT cells in hyperergy (i.e., hypersensitivity pneumonitis or sarcoidosis) (Katchar et al., 2005; Korosec et al., 2007). NKT cells (thymic origin) recognized endogenous and bacterial glycolipids presented by CD1d. Interestingly, coincidence of absolute number of NK and NKT cells and clinical status (IgM, β2M, FVC, PET findings) was observed (Figures 1, 2). Contrary to IVIG (Paquin-Proulx et al., 2013) the rituximab therapy at low and standard dose alleviated NKT cell level (Figure 2) but high level of T cell activation observed here and previously (Paquin-Proulx et al., 2013) was not normalized by RTx treatment (Table 1). The first explanation is the fact that low Treg levels are important to control immune responses and prompt persistent immune activation. On the other hand, low RTx dose causes more intensive CD38 expression than the higher (i.e., 350 mg/m^2^) dose, but without infectious complications observed during the third cycle of the standard dose. Antigen-specific interferon-releasing and CD38 expression (Table 1), both indicative of cellular immune activation, were associated with better course of the disease. It corresponds with last findings: expression of CD38 on tumor-infiltrating T cells correlates with longer survival of patients with carcinoma (Garnelo et al., 2017).

Vigorous CMV-specific IFNγ T cell response and high expansion of late CD57-positive CMV-specific effector CD8 (+) T-cell subset are modified by rituximab therapy (Table 1). Interferon gamma release after CMV epitope stimulation was also blocked by RTx, but the 375 mg/m^2^ dose blockade is widespread (polyclonal), since the dose is able to block post-mitogenic IFN release as well as specific immunity to latent VZV and *Streptococcus pneumoniae*. It is crucial that rituximab therapy blocks B cells but it may be the source of total lymphopenia and may affect adaptive humoral and cellular (i.e., CD8-dependent) response as shown in Table 1 by pentamer analysis. The latest findings in an animal model show that CD8^+^ T cells play an essential role in anti-CD20-mediated tumor regression but the blockade of regulatory T cells by CTLA-4 can synergize with anti-CD20 (Ren et al., 2017). Such CD8 cellular cytotoxicity is MHC class I-dependent. High serum level of MHC I invariant chain-β2M (Figure 1) as well as CD8 and NKT cells fluctuation were observed here (Figure 2). Interestingly, rituximab therapy causes a significant increase of both populations of T cells. The observation spotlights additional, dose-independent immunotherapeutic mechanism of rituximab therapy. Contrary to the animal model (Ren et al., 2017), the simultaneous Treg and B cells decrease observed here after low and standard dose of rituximab prompt further use of rituximab monotherapy rather than in combination with ipilimumab. Therefore, CVID and other immunodeficiency require a different immunotherapeutic approach. The immune dysregulation and decrease of Treg as a hallmark of CVID (Paquin-Proulx et al., 2013) was observed during LIP progression (Table 1; Zdziarski et al., 2017) and later Treg deficiency was not alleviated after RTx therapy. It is even intensified (Figure 2). Furthermore, such blockade of Treg can synergize with anti-CD20 treatment in antitumor activities by increase of T cell activation (i.e., increase CD38 expression, see Table 1). Interestingly, adaptive immune response-related resistance, not studied in human and adaptive immunity, has long been underestimated (Ren et al., 2017).

The recurrent therapy with a low dose (150 mg/m^2^) did not influence the incidence of infections, probably because of compensatory increase in innate immune response. Innate immune response is also disturbed in CVID: the previous study showed reduction in absolute numbers of invariant NKT (Trujillo et al., 2011; Paquin-Proulx et al., 2013) and NK (Trujillo et al., 2011) cells in patients with CVID. Innate immune responses are disturbed in some CVIDs, also observed here, but they improved after low dose rituximab therapy (Figure 2). This observation shows such an effect of rituximab for the first time. Furthermore, the low dose is more pleiotropic – it affects NKT (CD3+56+) and NK (CD16+56+) cell count but the standard dose –NK only (Figure 2 and Table 1 – NK/NKT ratio). Physiologically absolute NK and CD3+56+ NKT number and percentage are comparable (Zdziarski et al., 2016) so they reflect marrow and thymic abnormalities, respectively. NKT cells are a minor subpopulation of T cells, so they are of thymic origin, but NK cells development occurs extrathymically, mainly in the bone marrow. Furthermore, our patient showed lower NK level and that is the sign of LIP progression (Table 1). Low NK level (Trujillo et al., 2011) may be the result of marrow abnormalities in CVID. On the contrary: after a low dose of rituximab the decrease of T cells was observed and it was normalized after the use of the standard dose. Indeed, the thymus physiologically contains epithelial cells and thymocytes but B and plasmacytoid cells are also present. The influence of low level of Treg observed here and the latest finding that rituximab, at the concentration 5 × 10^4^μg/l (50 mg/l) induces the improvement of NKT activity via STAT and MAPK/ERK signaling pathways (Deng et al., 2015), may be – to some degree – the explanation of our data. In patients with interstitial lung disease, the FOXP3 (+)/CD3 (+) cell ratio and established fibrosis (EF) score were inversely correlated as described previously (Shimizu et al., 2010). Development of pulmonary fibrosis with respiratory insufficiency may ensue in LIP. On the other hand, our observation of FVC normalization with low level of Treg (Figure 2) and increase of activated CD38+ T cells were observed (Table 1). Our observation of rituximab monotherapy with a low dose prompts further use of such regimen in clinical practice. Pharmacovigilance is the second cause for low dose use. Progressive LPD with high lymphocyte or lymphoplasmacytoid cell burden (antigen) together with a high dose of specific antibody (i.e., rituximab) induce serum sickness and severe complications, especially tumor lysis syndrome as well as vasculopathy and interstitial pneumonitis as described elsewhere (Burton et al., 2003; Lioté et al., 2010). Such post-rituximab serum sickness together with the observed here initial IgM paraproteinemia (Figure 1) and hyperviscosity may be the cause of severe microvascular complication as well as pneumonitis. Severe hypergammaglobulinemia is described as a risk factor of such complications (Finger and Scheinberg, 2007; Zdziarski et al., 2017). Interestingly, serum sickness was observed only when the standard dose was used in monotherapy of immune thrombocytopenia but one patient developed an interstitial pneumonia nearly 1 month after the fourth administration of a low dose (i.e., 100 mg/m^2^) of rituximab (Zaja et al., 2012). Therefore, initial period of rituximab therapy of LIP shows very delicate risk-benefit balance. On the contrary to our patient with CVID and low B cells count (i.e., CD20+ 4,7% of lymphocytes and absolute count 72–120), patients in Burton’s report showed NHL with lymphocyte count of 44,900 per cubic millimeter. Most of patients with pulmonary complications reviewed elsewhere (Lioté et al., 2010) received rituximab in NHL, sometimes due to autoimmune indications, but unfortunately primary immunodeficiency with immune dysregulation and LIP has not been described previously. Severe LPD in CVID is sometimes reported with rituximab therapy but as a part of HSCT (Wehr et al., 2015). Furthermore, rituximab is very seldom used in monotherapy. The same interaction may be part of current clinical trial (NCT02789397^2^): rituximab is used in a standard dose with azathioprine. Therapeutic regimen of rituximab with multiple chemotherapy for NHL is also used in combination with glucocorticoids (CHOP with 100 mg of prednisone) and with preemptive growth factors, known proinflammatory and granulomatous-inducing mediators. Supportive G-CSFs are routinely used in primary or secondary prophylaxis of febrile neutropenia at each cycle. Strict rituximab-induced pneumonitis is therefore difficult to assess.

Presented here pleiotropic immunomechanism, rational and pharmacoeconomic ground for such therapy in LIP and other restrictive granulomatous interstitial lung diseases, is really the area of science in the future. The immune dysregulation and LPD progression described previously in LIP (Zdziarski et al., 2017), sometimes lymphoma, were the first indication for HSCT in majority of patients with CVID (Wehr et al., 2015). At present, transplantation is the only possible curative therapy (Rizzi et al., 2011).

## Conclusion

Normalization of high percentage of CMV pp65-specific CTL, increase of FVC, regression of radiographic and PET abnormalities several years after LIP onset indicate that lymphocytic infiltration of the alveolar interstitium and pulmonary defect are reversible, contrary to COPD. It may be monitored by spirometric (FVC), biochemical (β2M) serological (IgM paraproteinemia) parameters at little cost.

Glucocorticoid and IVIG therapy for LIP is ineffective but rituximab at a low dose (150 mg/m^2^) appears to be a good therapeutic regimen because of the same effectiveness as standard dose (spleen size, FVC, remission period) and compensatory increase of innate immune response as well as T cell activation with CD38 expression. RTX reduces antibody producing precursor plasma cells and inhibits B and T cells interaction. Infections related to T cell immunodeficiency (as VZV observed here) are not infrequent during RTX treatment (Besada and Nossent, 2016). *S. pneumoniae* – opportunistic pathogen in humoral immunodeficiency is a particular obligation to be cautious in immunosuppressive LIP therapy. The compensatory increase of NK, NKT, CD38+T cells and decrease of Treg (Figure 2 and Table 1) after RTX dose 150 mg/m^2^ with the same effectiveness as 375 mg/m^2^ may be a therapeutic option in the future and would be an interesting area to study prospectively. LIP/LPD development in patients with primary immunodeficiency requires a different investigative approach to immunotherapy directed against regulatory T cells.

## Ethics Statement

The patient gave a written informed consent in accordance with the Declaration of Helsinki.

## Author Contributions

PZ contributed to conception and design, collection of clinical data, therapeutic regimen, and drafting and editing the manuscript. AG performed the laboratory and microbiological analysis and critically revised the manuscript. All authors read and approved the final manuscript.

## Conflict of Interest Statement

The authors declare that the research was conducted in the absence of any commercial or financial relationships that could be construed as a potential conflict of interest.

## Footnotes

1 Product monograph of MabThera (last updated May 24, 2018) http://www.ema.europa.eu/ema/index.jsp?curl=pages/medicines/human/medicines/000165/human_med_000897.jsp&murl=menus/medicines/medicines.jsp&mid=WC0b01ac058001d124 *Pneumocystis jirovecii pneumonia (PCP) prophylaxis is recommended for patients with granulomatosis with polyangiitis or microscopic polyangiitis during and following MabThera treatment, as appropriate.(…) *Mean pre-therapeutic antibody titers against a panel of antigens (Streptococcus pneumoniae, influenza A, mumps, rubella, varicella) were maintained for at least 6 months after treatment with MabThera.**

2 Clinical trial (NCT02789397). This phase II study will assess the effect of a treatment combination of Rituximab and azathioprine in patients with Granulomatous and Lymphocytic Interstitial Lung Disease (GLILD) compared to placebo, based on change in lung function at 18 months compared to baseline. The researchers will also assess if the drugs improved the quality of life.

## Abbreviations

BAL: bronchoalveolar lavage
BMLF1: BamHI-M leftward reading frame 1 (immediate-early nuclear EBV antigen)
CVID: common variable immunodeficiency
DLCO: diffusion capacity of lung for carbon monoxide
FVC: forced vital capacity
HSCT: hematopoietic stem cell transplantation
IVIG: intravenous immunoglobulin infusions
LIP: lymphoid interstitial pneumonia/pneumonitis
LMP1: EBV latent membrane protein 1
LPD: lymphoproliferative disease
MALDI-TOF: matrix-assisted laser desorption ionization-time of flight mass spectrometry
NHL: Non-Hodgkin Lymphoma
NK: natural killer cell
NKT: natural killer T cell
PET: positron emission tomography
Quantiferon: gamma interferon-releasing assay
SUV: standardized uptake value
